# Development and validation of the AI literacy, risk perception, and academic confidence questionnaire for Chinese pre-service teachers

**DOI:** 10.1371/journal.pone.0353837

**Published:** 2026-07-16

**Authors:** Zeyu Zhang, Xiaomei Lu, Guochao Xiao, Xinde Wang

**Affiliations:** 1 Judicial Police College, Xinjiang University of Political Science and Law, Tumxuk, China; 2 Medical Appraisal Center, Sihong Hospital, Suqian, China; 3 School of Education and Psychology, Hubei Engineering University, Xiaogan, China; 4 School of Educational Science, Xinjiang Normal University, Urumqi, China; Ajman University, UNITED ARAB EMIRATES

## Abstract

Artificial intelligence (AI) is becoming increasingly relevant to teacher education, yet evidence remains limited on how pre-service teachers’ AI literacy, risk perception, and academic confidence can be assessed within a coherent but multidimensional framework. This study examined the psychometric properties of the AI Literacy, Risk Perception, and Academic Confidence Questionnaire (AIRPAC-Q) among Chinese pre-service teachers. A cross-sectional survey was conducted with 528 participants recruited from teacher education programmes in China. The sample was randomly divided into an exploratory factor analysis (EFA) subsample (n = 258) and a confirmatory factor analysis (CFA) subsample (n = 270). Psychometric evaluation included expert-based content validation, pilot refinement, EFA, CFA, reliability testing, convergent and discriminant validity, concurrent validity, and known-group validity analyses. The final questionnaire retained 14 items across three complementary dimensions: AI literacy, risk perception, and academic confidence. Expert ratings showed acceptable content validity (I-CVI = 0.83–1.00; S-CVI/Ave = 0.94). The hypothesized three-factor model showed an acceptable fit to the data (χ² = 146.32, df = 74, χ²/df = 1.98, CFI = 0.952, TLI = 0.941, RMSEA = 0.060, SRMR = 0.047) and outperformed two-factor and one-factor alternatives. Cronbach’s α values ranged from 0.82 to 0.88, composite reliability values ranged from 0.83 to 0.89, and average variance extracted values ranged from 0.56 to 0.59. Participants with prior AI use experience scored higher on AI literacy and academic confidence but slightly lower on risk perception than those without such experience. These findings support the AIRPAC-Q as a context-specific multidimensional tool for assessing competence, caution, and confidence in AI-supported teacher education.

## Introduction

Artificial intelligence (AI) is becoming increasingly visible in education, not only as a topic of public discussion but also as a practical tool used in teaching, learning, assessment, feedback, and academic decision-making. Educators and students now encounter AI-supported systems in activities such as automated feedback, content generation, information search, lesson preparation, and classroom support. The question is therefore no longer whether AI will influence education, but how educational institutions should prepare future teachers to understand, evaluate, and use AI appropriately. This issue is especially important in teacher education, where pre-service teachers are expected to develop both the technical awareness and the pedagogical judgment needed to work in AI-supported learning environments [1–3].

Existing studies have often focused on only one part of this broader picture. Some research has examined students’ or teachers’ attitudes toward AI-supported learning and feedback, emphasizing perceived usefulness, convenience, or motivational value [1]. Other work has investigated teacher readiness for AI in classrooms through dimensions such as knowledge, attitudes, and practices [3]. More recently, research on prospective teachers has begun to examine AI literacy and acceptance of generative AI together, showing that the ability to understand AI is related to willingness to adopt it in educational contexts [2]. International evidence on students’ early reactions to ChatGPT also suggests that perceptions of AI are mixed and multidimensional, involving opportunities, uncertainty, ethical concerns, and contextual differences rather than simple enthusiasm or rejection [4]. Taken together, these studies suggest that AI-related educational behavior is shaped not only by access to technology, but also by how future educators understand AI, evaluate its possible consequences, and maintain confidence in academic judgment when AI tools are available.

In the present study, the AIRPAC-Q is therefore conceptualized as a multidimensional profile of AI-related educational functioning rather than as a single higher-order latent trait. AI literacy represents the competence component, risk perception represents the evaluative-caution component, and academic confidence represents the self-belief and judgment component. These dimensions are expected to be related because they all concern how pre-service teachers engage with AI-supported academic tasks, but they are not assumed to be interchangeable indicators of one underlying construct. This position is consistent with broader educational technology perspectives in which technology use depends on perceived capability, perceived value or utility, perceived costs and risks, and confidence in one’s own learning decisions. The scale is thus intended to describe a structured configuration of competence, caution, and confidence, not to collapse them into a single readiness score.

AI literacy has become an increasingly important concept in research on digital learning and teacher education. In educational settings, AI literacy extends beyond basic awareness of AI tools and includes the ability to understand how such systems function, evaluate their outputs critically, and use them appropriately for academic and pedagogical purposes. Recent work with pre-service teachers further suggests that AI literacy should be understood not simply as familiarity with tools, but as a multidimensional capacity involving technical understanding, critical evaluation, and contextually appropriate educational use [5,6]. This broader view is consistent with emerging teacher-focused work showing that AI literacy in education includes judging the suitability, limitations, and pedagogical implications of AI-generated outputs rather than merely operating AI-enabled tools [7,8]. For pre-service teachers, such literacy is especially important because they are expected not only to use AI in their own learning but also to develop the professional judgment needed to evaluate AI-supported practices in future teaching.

A second construct relevant to AI-supported learning is risk perception. The use of AI in education may be accompanied by concerns about inaccuracy, overreliance, bias, privacy, academic integrity, and the possible erosion of independent judgment. In this sense, risk perception reflects not resistance to innovation per se, but a learner’s awareness of uncertainty, potential negative consequences, and the need for caution when engaging with AI tools. Recent higher education research on generative AI also suggests that risk-related concerns remain consequential for educational technology use, as perceived risks and avoidance tendencies can shape whether students approach AI as an empowering resource or a source of uncertainty and potential harm [9]. For pre-service teachers, this dimension may be particularly salient because they are preparing for professional roles in which decisions about technology use are not merely personal choices, but also carry pedagogical and ethical implications.

A third construct that deserves closer attention is academic confidence. In the AIRPAC-Q, academic confidence refers to pre-service teachers’ perceived ability to make learning-related judgments, regulate academic tasks, and remain agentic when AI tools are present. This differs from AI literacy, which concerns understanding, evaluating, and using AI systems, and it also differs from general self-efficacy, which is a broader belief about capability across tasks and situations. Academic confidence is narrower and more context-specific: it concerns confidence in managing academic decision-making in AI-supported learning environments. This distinction matters because students may understand AI tools but still feel uncertain about how much to rely on them, or conversely may feel confident in academic judgment while remaining cautious about the limitations of AI-generated outputs. Recent work with preservice teachers indicates that confidence in AI-related learning contexts can develop alongside growing knowledge, while concerns and uncertainty may still persist, suggesting that confidence should not be treated as reducible to either general self-efficacy or simple technology optimism [10].

Teacher education provides an especially important context for studying these issues. Recent studies in teacher education have examined pre-service teachers’ AI-related acceptance, AI-TPACK, and course participation, indicating growing interest in their readiness for AI-supported practice, but these studies have typically focused on intention, acceptance, or competence beliefs rather than on an integrated measurement of competence, caution, and confidence [11]. Similarly, recent self-assessment approaches to teachers’ AI literacy have provided useful evidence about competence dimensions, but they do not fully capture how perceptions of risk and confidence in academic judgment coexist within the same measurement framework [8]. The choice of AI use intention as an external criterion is also supported by recent evidence showing that AI literacy is meaningfully associated with students’ intentions to engage with generative AI tools in educational settings [12]. Existing studies have therefore offered valuable but still fragmented evidence, making it difficult to assess how pre-service teachers balance understanding, caution, and self-regulatory confidence in AI-supported learning environments.

Accordingly, the present study addressed three research questions. First, does the AIRPAC-Q show a theoretically interpretable three-factor structure corresponding to AI literacy, risk perception, and academic confidence among Chinese pre-service teachers? Second, does the instrument provide acceptable evidence of internal consistency, convergent validity, and discriminant validity? Third, are AIRPAC-Q subscale scores meaningfully associated with AI use intention in future teaching and with prior AI use experience? By answering these questions, the study aims to provide a context-specific instrument for descriptive assessment and future programme evaluation in teacher education, while avoiding the assumption that the three dimensions form a single total score.

## Methods

### Study design and participants

This study used a cross-sectional survey design to develop and validate the AIRPAC-Q among Chinese pre-service teachers. Participants were recruited from undergraduate teacher education programmes at four public universities in western and eastern China through classroom-based invitations and supervised online distribution. The participating institutions offered teacher preparation programmes in areas such as language education, mathematics education, science education, and educational technology. The recruitment strategy was intended to include more than one institutional and regional context, but it was not designed to produce a nationally representative sample. Students were eligible if they were enrolled in a pre-service teacher education programme and provided informed consent prior to participation.

Data were collected between 15 October and 28 December 2025 using Wenjuanxing, a widely used Chinese online survey platform. A total of 552 questionnaires were returned. After excluding cases with substantial missing data, patterned responding, and implausibly short completion times, 528 valid questionnaires were retained for analysis. The final sample included 146 men (27.7%) and 382 women (72.3%). With respect to year of study, 112 (21.2%) participants were in Year 1, 138 (26.1%) in Year 2, 149 (28.2%) in Year 3, and 129 (24.4%) in Year 4. In addition, 318 participants (60.2%) reported prior experience using AI tools for learning, whereas 210 (39.8%) reported no such experience. Teaching practicum experience was reported by 241 participants (45.6%).

**Fig 1:**
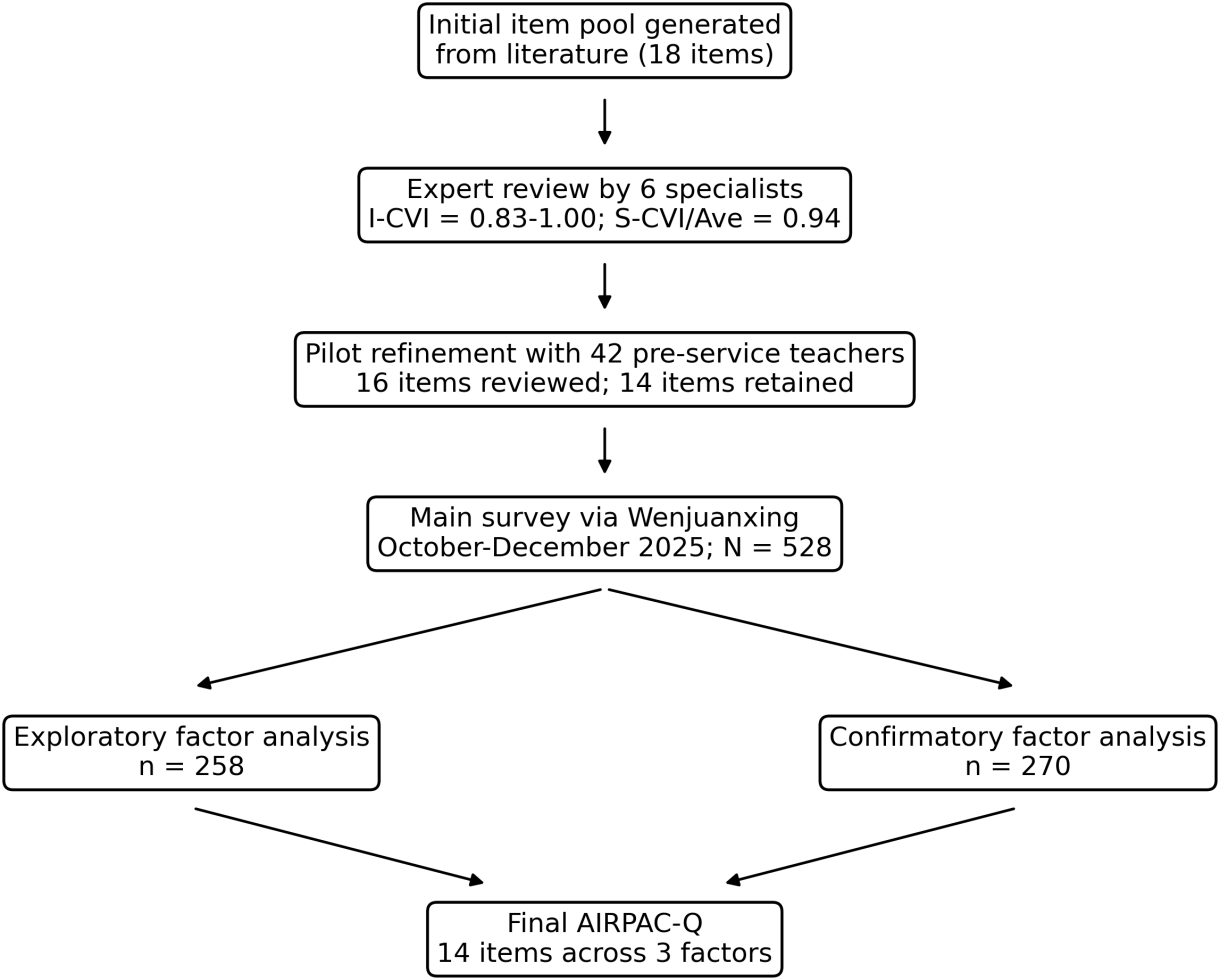
Workflow for the development and validation of the AIRPAC-Q. The initial item pool was generated from the literature, reviewed by six experts, refined through pilot testing, and then administered to a main sample of Chinese pre-service teachers. The full sample was randomly split into an exploratory factor analysis subsample and a confirmatory factor analysis subsample. The final questionnaire retained 14 items across three factors. Note. AIRPAC-Q = AI Literacy, Risk Perception, and Academic Confidence Questionnaire; EFA = exploratory factor analysis; CFA = confirmatory factor analysis.

To support scale development and validation, the total sample was randomly divided into two independent subsamples. The first subsample (n = 258) was used for exploratory factor analysis (EFA), and the second subsample (n = 270) was used for confirmatory factor analysis (CFA). The overall scale development and validation process is presented in Fig 1.

### Instrument development

The AIRPAC-Q was developed to assess three related but distinct dimensions of AI-related educational functioning among pre-service teachers: AI literacy, risk perception, and academic confidence. Items were drafted in Chinese for administration and translated into English for reporting in this manuscript. Item generation was informed by prior literature on AI literacy in education, educational risk perception, academic confidence, trust in automated systems, and technology-related judgment [2,13–20]. The initial pool contained 18 items, with six candidate items written for each of the three target constructs. The wording of each item was reviewed to ensure that it referred to AI-supported learning or educational contexts rather than to general technology use.

The preliminary item pool was reviewed by six experts with backgrounds in educational psychology, teacher education, educational technology, and psychometrics. The expert panel included scholars or senior instructors with experience in teacher education, scale development, digital learning, or educational measurement. Experts evaluated each item in terms of clarity, relevance, and representativeness for the intended construct. Based on these evaluations, item-level content validity indices (I-CVI) and the scale-level content validity index (S-CVI/Ave) were calculated. Two candidate items were removed after expert review: one risk perception item was judged to be double-barreled because it combined privacy, bias, and academic integrity in one statement, and one academic confidence item was judged to overlap too strongly with general self-efficacy rather than AI-supported academic judgment. The I-CVI values for the retained items ranged from 0.83 to 1.00, and the S-CVI/Ave for the retained full scale was 0.94, indicating satisfactory content validity [21,22].

The remaining 16 items were pilot tested with 42 pre-service teachers who were not included in the main validation sample. The pilot test examined readability, interpretability, response clarity, and item redundancy. Participants were also invited to report unclear wording after completing the questionnaire. Minor wording revisions were made to improve the specificity of references to AI-supported learning. Two additional items were removed after pilot refinement: one risk perception item was redundant with the retained caution-related items, and one academic confidence item showed weak discrimination and was interpreted by several respondents as general confidence rather than confidence in AI-supported academic tasks. This process resulted in the final 14-item questionnaire. A summary of item sources, expert feedback, pilot revisions, and content validity indices is provided in S2 Table.

The final instrument included three subscales. AI literacy (6 items) assessed perceived understanding, critical evaluation, and appropriate use of AI tools in educational settings. These items were informed primarily by prior conceptualizations of AI literacy as knowledge, evaluation, and appropriate use of AI systems in educational contexts [13,23]. A sample item was, “I understand the basic functions of AI tools used in education.” Risk perception (4 items) assessed perceived uncertainty, caution, and possible negative consequences associated with AI use in education. These items were informed by classic and applied work on perceived uncertainty, consequence severity, and caution in decision contexts [14,15,20]. A sample item was, “The use of AI in education may involve important risks.” Academic confidence (4 items) assessed confidence in managing learning tasks and making academic judgments in AI-supported learning environments. These items were informed by prior work on academic behavioural confidence and learning-related judgment in higher education [16], but were rewritten to refer specifically to academic judgment when AI tools are involved. A sample item was, “I feel confident in making academic judgments in AI-supported learning environments.”

All AIRPAC-Q items were rated on a five-point Likert-type scale ranging from 1 (strongly disagree) to 5 (strongly agree). Subscale scores were recommended to be calculated as mean scores rather than as a single total score because the instrument is multidimensional. The full questionnaire and scoring instructions are provided in S1 Appendix, and the expert review summary with item-level and scale-level content validity indices is provided in S2 Table.

### External measure for concurrent validity

To examine concurrent validity, participants also completed a four-item scale assessing intention to use AI in future teaching. The items were adapted from prior technology acceptance research with pre-service teachers and from recent work on candidate teachers’ acceptance of generative AI [2,24]. A sample item was “I intend to use AI tools in my future teaching when appropriate.”

Items were rated on the same five-point Likert-type scale as the AIRPAC-Q, and higher scores indicated stronger intention to use AI in educational practice. This external measure was selected because it was theoretically expected to correlate positively with AI literacy and academic confidence and to show weaker or inverse associations with risk perception. A sample item is reported in the main text, and the source studies from which the items were adapted are cited accordingly.

### Procedure

Participants were informed that the study focused on AI-related educational experiences and learning judgments among pre-service teachers. The questionnaire was completed anonymously either in classroom settings or through a secure online platform. To reduce evaluation apprehension, participants were assured that there were no right or wrong answers and that responses would be used only for research purposes. Only participants who clicked “I agree to participate” were able to proceed to the questionnaire.

### Statistical analysis

Data analysis was conducted using SPSS 27.0 and AMOS 26.0. Descriptive statistics were first calculated for the full sample, including frequencies, means, and standard deviations. Item quality was examined using corrected item-total correlations and item distributions.

The psychometric evaluation proceeded in several steps. First, the EFA subsample was used to assess the underlying factor structure of the questionnaire. The suitability of the data for factor analysis was examined using the Kaiser-Meyer-Olkin (KMO) measure and Bartlett’s test of sphericity. Exploratory factor analysis was conducted using principal axis factoring with oblimin rotation, given the expected correlations among the latent dimensions. Factor retention was guided by eigenvalues, scree plot inspection, communalities, and conceptual interpretability. Items with primary loadings below 0.40, low communalities, or substantial cross-loadings were considered for removal. Alternative factor solutions were inspected before retaining the final structure.

Second, the CFA subsample was used to test the hypothesized three-factor structure. Model fit was evaluated using the chi-square statistic divided by degrees of freedom (χ²/df), the comparative fit index (CFI), the Tucker-Lewis index (TLI), the root mean square error of approximation (RMSEA), and the standardized root mean square residual (SRMR). To assess model distinctiveness, the three-factor model was compared with more parsimonious two-factor and one-factor alternatives. Standardized factor loadings were examined, and model modifications were not introduced unless theoretically justified.

Third, internal consistency was assessed using Cronbach’s α, while convergent validity was examined through composite reliability (CR) and average variance extracted (AVE). Discriminant validity was evaluated using correlations among the three factors and comparison of the square roots of the AVE values with the inter-factor correlations [25,26]. Because the AIRPAC-Q is conceptualized as multidimensional, subscale scores rather than a total score were used in validity analyses.

Fourth, the potential influence of common-method variance was examined using Harman’s single-factor test and by considering the fit of the one-factor CFA model [27]. These procedures were treated as limited diagnostics rather than as definitive tests, because all AIRPAC-Q items were self-reported and collected at the same time. Concurrent validity was examined using Pearson correlations between AIRPAC-Q subscale scores and AI use intention in future teaching. Known-group validity was explored by comparing participants with and without prior AI use experience using independent-samples t tests. Mean differences, 95% confidence intervals, and Cohen’s d values were reported for these comparisons. A two-tailed p < 0.05 was considered statistically significant throughout.

### Ethics statement

This study was reviewed and approved by the Research Office of Xinjiang University of Political Science and Law (Approval No. XJPL-KYC-2025–001). Participation was voluntary and anonymous. Written informed consent was obtained electronically from all participants before they began the online survey. The questionnaire was administered via Wenjuanxing, a widely used online survey platform in China, between 15 October 2025 and 28 December 2025. No personally identifiable information was collected. All data were stored securely and used solely for research purposes.

## Results

### Sample characteristics

A total of 528 valid questionnaires were included in the analysis. The sample was predominantly female (72.3%), reflecting the gender composition of teacher education programmes in the participating institutions. More than half of the participants (60.2%) reported prior experience using AI tools in learning contexts. Detailed sample characteristics are presented in Table 1.

**Table 1:** Sample characteristics of the pre-service teacher participants.

Variable	Category	n	%
Gender	Male	146	27.7
	Female	382	72.3
Grade	Year 1	112	21.2
	Year 2	138	26.1
	Year 3	149	28.2
	Year 4	129	24.4
Prior AI use experience	Yes	318	60.2
	No	210	39.8
Teaching practicum experience	Yes	241	45.6
	No	287	54.4

Note. N = 528. Values are presented as n (%). Percentages may not total 100 because of rounding.

### Content validity

Expert ratings indicated satisfactory content validity for the AIRPAC-Q. The item-level content validity indices (I-CVI) for the retained items ranged from 0.83 to 1.00, and the scale-level content validity index (S-CVI/Ave) was 0.94. Expert comments mainly concerned reducing conceptual overlap, avoiding double-barreled wording, and ensuring that academic confidence items referred specifically to AI-supported learning rather than to general self-efficacy. These results suggested that the retained items were considered relevant and representative of the intended constructs by the expert panel. Detailed expert ratings, item sources, and revision decisions are reported in S2 Table.

### Item analysis and exploratory factor analysis

Item-level descriptive statistics indicated that the 14 retained items showed acceptable variability, with mean scores ranging from 3.39 to 3.91 and standard deviations ranging from 0.76 to 0.95. Corrected item-total correlations ranged from 0.53 to 0.68, suggesting satisfactory item discrimination. Communalities ranged from 0.46 to 0.67, indicating that the retained items were adequately represented by the extracted factors.

The data were suitable for factor analysis, as indicated by a KMO value of 0.89 and a significant Bartlett’s test of sphericity (χ² = 2874.56, df = 91, p < 0.001). Exploratory factor analysis supported a three-factor structure corresponding to AI literacy, risk perception, and academic confidence. A two-factor solution was not retained because it combined competence- and confidence-related items in a way that was less consistent with the conceptual distinction between AI literacy and academic confidence. A four-factor solution was also inspected but produced a weak and difficult-to-interpret split within the AI literacy items. As shown in Table 2, all retained items loaded on their intended factors, with primary factor loadings ranging from 0.68 to 0.82 and no substantial cross-loadings. The three-factor solution accounted for 62.8% of the total variance.

**Table 2:** Item descriptives, corrected item-total correlations, communalities, and exploratory factor loadings for the AIRPAC-Q.

Item	Mean	SD	CITC	h²	AI literacy	Risk perception	Academic confidence
AIL1	3.84	0.83	0.63	0.56	0.74	—	—
AIL2	3.76	0.86	0.66	0.61	0.78	—	—
AIL3	3.58	0.91	0.61	0.53	0.73	—	—
AIL4	3.65	0.88	0.68	0.66	0.81	—	—
AIL5	3.42	0.95	0.58	0.48	0.69	—	—
AIL6	3.51	0.89	0.64	0.58	0.76	—	—
RP1	3.48	0.87	0.55	0.50	—	0.71	—
RP2	3.62	0.82	0.57	0.55	—	0.74	—
RP3	3.91	0.76	0.61	0.63	—	0.79	—
RP4	3.57	0.85	0.53	0.46	—	0.68	—
AC1	3.71	0.79	0.60	0.58	—	—	0.76
AC2	3.64	0.81	0.66	0.67	—	—	0.82
AC3	3.39	0.92	0.54	0.48	—	—	0.69
AC4	3.56	0.86	0.59	0.53	—	—	0.73

Note. EFA was conducted using principal axis factoring with oblimin rotation. KMO = 0.89; Bartlett’s test of sphericity, χ² = 2874.56, df = 91, p < 0.001. The three-factor solution explained 62.8% of the total variance. Communality values (h²) indicate the proportion of variance in each item accounted for by the extracted factors. CITC = corrected item-total correlation. Only primary factor loadings of 0.40 or above are shown; all cross-loadings were below the conventional cutoff.

### Confirmatory factor analysis and model comparison

Confirmatory factor analysis was conducted to test the hypothesized three-factor structure. The three-factor model showed an acceptable fit to the data (χ² = 146.32, df = 74, χ²/df = 1.98, CFI = 0.952, TLI = 0.941, RMSEA = 0.060, SRMR = 0.047). Standardized factor loadings were 0.69–0.81 for AI literacy, 0.68–0.79 for risk perception, and 0.69–0.82 for academic confidence; all loadings were statistically significant. No correlated error terms or post hoc model modifications were introduced. As shown in Table 3, the three-factor model also outperformed the two-factor and one-factor alternatives, supporting the distinctiveness of AI literacy, risk perception, and academic confidence.

**Table 3:** Confirmatory factor analysis and model comparison for the AIRPAC-Q.

Model	χ²	df	χ²/df	CFI	TLI	RMSEA	SRMR
Three-factor model	146.32	74	1.98	0.952	0.941	0.060	0.047
Two-factor model	279.48	76	3.68	0.842	0.807	0.099	0.086
One-factor model	431.77	77	5.61	0.731	0.681	0.131	0.112

*Note. CFI = comparative fit index; TLI = Tucker-Lewis index; RMSEA = root mean square error of approximation; SRMR = standardized root mean square residual.*

**Fig 2:**
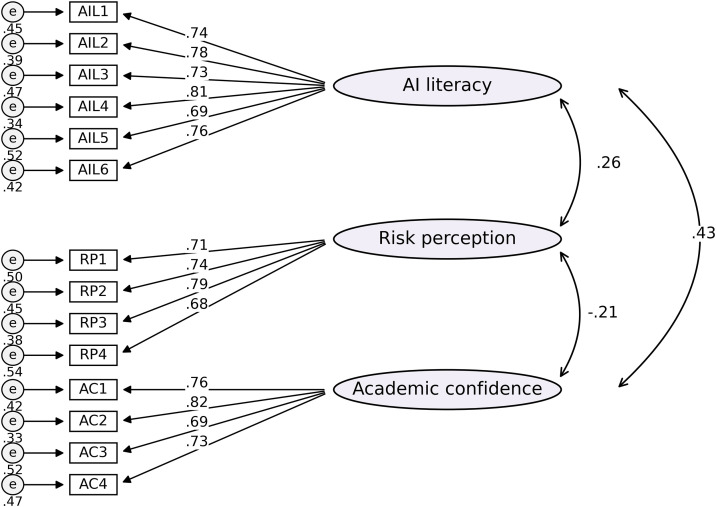
Standardized confirmatory factor analysis model of the AIRPAC-Q. Rectangles represent observed items, circles represent error terms, and ellipses represent latent constructs. Standardized factor loadings are shown on the single-headed arrows, and correlations among the latent constructs are shown on the double-headed arrows. Note. AIRPAC-Q = AI Literacy, Risk Perception, and Academic Confidence Questionnaire.

The two-factor model showed a poorer fit (χ² = 279.48, df = 76, χ²/df = 3.68, CFI = 0.842, TLI = 0.807, RMSEA = 0.099, SRMR = 0.086), and the one-factor model showed the weakest fit (χ² = 431.77, df = 77, χ²/df = 5.61, CFI = 0.731, TLI = 0.681, RMSEA = 0.131, SRMR = 0.112). These comparisons indicate that the AIRPAC-Q is better represented as three related subscales than as a single undifferentiated readiness measure. Model comparison results are presented in Table 3, and the standardized CFA model is shown in Fig 2.

### Reliability and convergent validity

The AIRPAC-Q demonstrated acceptable to good internal consistency across all three subscales. Cronbach’s α was 0.88 for AI literacy, 0.82 for risk perception, and 0.84 for academic confidence. Composite reliability values ranged from 0.83 to 0.89, exceeding conventional adequacy thresholds. Average variance extracted values ranged from 0.56 to 0.59, indicating acceptable convergent validity for all three constructs. These results are reported in Table 4.

**Table 4:** Reliability and convergent validity of the AIRPAC-Q.

Construct	Cronbach’s α	CR	AVE
AI literacy	0.88	0.89	0.58
Risk perception	0.82	0.83	0.56
Academic confidence	0.84	0.85	0.59

Note. Cronbach’s α = Cronbach’s alpha; CR = composite reliability; AVE = average variance extracted.

### Discriminant validity

Descriptive statistics, inter-factor correlations, and discriminant validity estimates are shown in Table 5. AI literacy was positively correlated with risk perception (r = 0.26, p < 0.01) and academic confidence (r = 0.43, p < 0.01). Risk perception was negatively correlated with academic confidence (r = −0.21, p < 0.01). As shown in Table 5, the square roots of the average variance extracted for all three constructs exceeded the corresponding inter-factor correlations, providing further support for discriminant validity.

**Table 5:** Descriptive statistics, inter-factor correlations, and discriminant validity of the AIRPAC-Q.

Variable	Mean	SD	1	2	3
1. AI literacy	3.63	0.68	0.76		
2. Risk perception	3.64	0.59	0.26**	0.75	
3. Academic confidence	3.58	0.63	0.43**	−0.21**	0.77

Note. Diagonal values in bold are the square roots of the average variance extracted (AVE). Off-diagonal values are Pearson correlations among the latent constructs. p < 0.01.

### Common-method bias

To assess the potential influence of common-method variance, Harman’s single-factor test was conducted as a preliminary diagnostic. The first unrotated factor accounted for 31.4% of the total variance, which suggested that no single general factor dominated the EFA solution. The poor fit of the one-factor CFA model provided additional evidence that the data were not adequately represented by one common factor. However, these results should be interpreted cautiously because Harman’s test and one-factor model comparisons cannot rule out common-method variance in a cross-sectional self-report study.

### Concurrent validity

Concurrent validity was examined by correlating AIRPAC-Q subscale scores with AI use intention in future teaching. AI literacy showed a moderate positive correlation with the criterion variable (r = 0.44, p < 0.001), and academic confidence also showed a positive correlation (r = 0.39, p < 0.001). Risk perception showed a weaker negative association (r = −0.17, p = 0.004). These results supported the concurrent validity of the scale with a theoretically related criterion and suggested that the AIRPAC-Q was meaningfully associated with relevant educational dispositions.

### Known-group validity

Known-group validity was examined by comparing participants with and without prior AI use experience. As shown in Table 6, participants with prior AI use experience scored significantly higher on AI literacy (M = 3.87, SD = 0.56) than those without such experience (M = 3.29, SD = 0.63; mean difference = 0.58, 95% CI [0.47, 0.69], t = 10.74, p < 0.001, Cohen’s d = 0.99). They also reported significantly higher academic confidence (M = 3.71, SD = 0.58) than participants without prior AI use (M = 3.42, SD = 0.66; mean difference = 0.29, 95% CI [0.18, 0.40], t = 5.18, p < 0.001, Cohen’s d = 0.47). By contrast, participants with prior AI use reported slightly lower risk perception (M = 3.34, SD = 0.61) than those without prior AI use (M = 3.52, SD = 0.57; mean difference = −0.18, 95% CI [−0.28, −0.08], t = −3.45, p = 0.001, Cohen’s d = −0.30).

**Table 6:** Known-group validity of the AIRPAC-Q by prior AI use experience.

Variable	Prior AI use (n = 318)	No prior AI use (n = 210)	Mean difference [95% CI]	t (p)	Cohen’s d
AI literacy	3.87 ± 0.56	3.29 ± 0.63	0.58 [0.47, 0.69]	10.74 (<0.001)	0.99
Risk perception	3.34 ± 0.61	3.52 ± 0.57	−0.18 [−0.28, −0.08]	−3.45 (0.001)	−0.30
Academic confidence	3.71 ± 0.58	3.42 ± 0.66	0.29 [0.18, 0.40]	5.18 (<0.001)	0.47

Note. Values are presented as mean ± standard deviation unless otherwise indicated. Mean differences were calculated as prior AI use minus no prior AI use. Group differences were examined using independent-samples t tests. CI = confidence interval; AI = artificial intelligence.

These findings provide additional support for the validity of the questionnaire, suggesting that the AIRPAC-Q is sensitive to meaningful group differences related to actual AI exposure in educational settings. The effect size was large for AI literacy, small-to-moderate for academic confidence, and small for risk perception, which is consistent with the interpretation that prior AI use is most closely related to familiarity and perceived capability, while caution may change more modestly with experience.

## Discussion

Conceptually, the AIRPAC-Q suggests that AI-related educational functioning in pre-service teachers is not a unidimensional readiness construct, but a structured configuration of competence, caution, and confidence. This revision explicitly treats the three dimensions as complementary components of AI-related educational functioning rather than as manifestations of a single higher-order latent trait. This framing helps reposition AI readiness in teacher education as a judgmental and developmental construct rather than as a purely attitudinal or adoption-oriented one.

The present study developed and validated the AIRPAC-Q as a multidimensional instrument for assessing AI-related educational dispositions among Chinese pre-service teachers. The findings supported a three-factor structure consisting of AI literacy, risk perception, and academic confidence, with acceptable evidence for content validity, structural validity, internal consistency, convergent validity, discriminant validity, concurrent validity, and known-group validity. These results suggest that AI-related readiness in teacher education is not adequately captured by competence-oriented measures alone. In this sense, the present findings extend recent work on pre-service teachers’ AI literacy and AI-related preparation by suggesting that competence-oriented views are incomplete unless they are considered alongside perceived risk and confidence in academic judgment [5,11]. Rather than treating AI-related readiness as a single positive disposition, the AIRPAC-Q frames it as a balance of competence, caution, and confidence.

The second major finding was that the AIRPAC-Q demonstrated acceptable psychometric quality across multiple validity indicators. The expert-based content validity results suggested that the retained items were judged to be relevant and representative of the intended constructs, while the EFA and CFA results supported the proposed three-factor structure. Importantly, the model comparison results and the moderate rather than very high correlation between AI literacy and academic confidence support the conceptual distinction between these dimensions. AI literacy concerns perceived understanding and evaluation of AI systems, whereas academic confidence concerns the learner’s perceived ability to make academic judgments and manage learning when AI tools are involved. The correlation between the two constructs is therefore theoretically expected, but it does not imply redundancy.

The concurrent validity results strengthen this interpretation. The AIRPAC-Q dimensions showed meaningful associations with an external criterion measure, suggesting that the scale is not only internally coherent but also related to a theoretically relevant educational disposition. AI literacy and academic confidence were positively associated with intention to use AI in future teaching, whereas risk perception showed a weaker negative association. This pattern suggests that perceived competence and academic self-belief may support future-oriented adoption intentions, while risk perception may function as a cautionary orientation rather than as simple opposition to AI.

The known-group validity findings provide additional practical value. Participants with prior AI use experience reported higher AI literacy and academic confidence than those without such experience, while also reporting slightly lower risk perception. The positive association between AI literacy and risk perception observed in the correlation analysis deserves particular attention. Although higher literacy might be expected to reduce perceived risk, it may also increase awareness of possible errors, bias, privacy concerns, and overreliance. In this sense, more knowledgeable pre-service teachers may not simply become less cautious; they may become better able to identify specific risks. Prior experience, by contrast, may increase familiarity and perceived controllability, which could partly explain the slightly lower risk perception among AI users. These findings support a nuanced interpretation in which literacy, experience, and caution do not move in a single direction.

The findings also have implications for teacher education, although these implications should be interpreted cautiously within the limits of an initial validation study. Teacher education programmes could use the AIRPAC-Q as a diagnostic or formative assessment tool to identify different profiles of AI-related development. For example, students with relatively high AI literacy but low risk perception may benefit from ethical reflection, source evaluation, and academic integrity activities. Students with high risk perception but low academic confidence may benefit from scaffolded practice, guided comparison of AI-generated and human-generated outputs, and opportunities to make independent judgments with instructor support. At the programme level, aggregated subscale profiles could inform curriculum design by showing whether training is overemphasizing technical familiarity while underdeveloping critical caution or judgment confidence.

Several limitations should be acknowledged. First, the study relied on a cross-sectional self-report design, and the findings therefore do not speak to longitudinal stability or causal relationships among the three constructs. Second, test-retest reliability and predictive validity were not examined; future research should determine whether AIRPAC-Q scores are temporally stable and whether they predict actual AI use, AI integration behavior, teaching readiness, or academic performance. Third, although Harman’s single-factor test and the one-factor CFA comparison suggested that no single factor dominated the data, these procedures cannot rule out common-method variance. The use of self-report items collected at one time point also raises the possibility of social desirability bias, especially because positive engagement with AI may increasingly be seen as normatively desirable in higher education. Fourth, the sample was drawn from Chinese pre-service teachers and was predominantly female, reflecting the gender composition of many teacher education programmes but limiting external validity. The meaning of risk perception may also vary across cultural and institutional contexts. Fifth, measurement invariance across gender, year of study, subject specialization, prior AI experience, and cultural groups remains to be established before the AIRPAC-Q can be used confidently for subgroup comparisons. Future research should also consider item response theory, bifactor modeling, and additional discriminant validity evidence to further evaluate the scale’s dimensional structure [28, 29].

The contribution of the AIRPAC-Q therefore lies not only in providing a new measurement tool, but also in offering a more integrated way of conceptualizing AI-related educational functioning in teacher education. The instrument may support future research on how Chinese pre-service teachers differ in their readiness to engage with AI, and it may provide a useful basis for descriptive assessment and programme evaluation in teacher education settings after further validation. The contribution of the AIRPAC-Q is comparable in spirit to recent PLOS ONE scale-development studies in education, which have emphasized context-specific instrument construction and validation, while differing in its focus on AI-related educational functioning among Chinese pre-service teachers [1,30]. At the same time, the present findings should be interpreted within the boundaries of the study design. Because the data were cross-sectional and self-reported, and because temporal stability, predictive validity, and measurement invariance were not tested, the AIRPAC-Q should be viewed as a psychometrically supported starting point rather than as a fully established universal measure.

## Supporting information

S1 Appendix Full questionnaire and scoring instructions for the AI Literacy, Risk Perception, and Academic Confidence Questionnaire (AIRPAC-Q). (DOCX)

S2 Table Item sources, expert review summary, pilot revision decisions, and content validity indices for the AIRPAC-Q items. (DOCX)
