# Supplementary material for: Development and validation of the AI literacy, risk perception, and academic confidence questionnaire for Chinese pre-service teachers
Source: PLoS One. 2026 Jul 16;21(7):e0353837. doi: 10.1371/journal.pone.0353837 (PMC13375133; doi:10.1371/journal.pone.0353837)
Supplement: S1 Appendix — (DOCX) [file pone.0353837.s001.docx]

**S1 Appendix. Full questionnaire and scoring instructions for the AI Literacy, Risk Perception, and Academic Confidence Questionnaire (AIRPAC-Q)**

# Title

AI Literacy, Risk Perception, and Academic Confidence Questionnaire (AIRPAC-Q)

# Instructions to participants

Below are a number of statements about your understanding of artificial intelligence (AI), your perceptions of possible risks related to AI in education, and your confidence when learning in AI-supported environments. Please indicate the extent to which you agree with each statement.

# Response scale

1 = strongly disagree

2 = disagree

3 = neither agree nor disagree

4 = agree

5 = strongly agree

# Scoring note

Subscale scores are recommended to be calculated as mean scores rather than as a single total score because the instrument is multidimensional.

# AI literacy (AIL)

AIL1. I understand the basic functions of AI tools used in education.

AIL2. I can distinguish between reliable and unreliable AI-generated information.

AIL3. I know that AI outputs may contain errors or bias.

AIL4. I am able to use AI tools appropriately to support my learning.

AIL5. I understand the limitations of AI in educational contexts.

AIL6. I can critically evaluate suggestions generated by AI systems.

# Risk perception (RP)

RP1. The use of AI in education may involve important risks.

RP2. AI-generated content may sometimes mislead learners.

RP3. I need to be cautious when relying on AI for academic tasks.

RP4. The inappropriate use of AI may have negative consequences for learning.

# Academic confidence (AC)

AC1. I feel confident in making academic judgments in AI-supported learning environments.

AC2. I can usually make effective learning decisions when using AI tools.

AC3. I feel capable of adapting to AI-related academic tasks.

AC4. I am confident in managing my learning even when AI tools are involved.

# Scoring procedures

The AIRPAC-Q is a multidimensional instrument with three subscales:

# Subscales

AI literacy (6 items): AIL1–AIL6

Risk perception (4 items): RP1–RP4

Academic confidence (4 items): AC1–AC4

# Recommended scoring approach

Calculate subscale scores as mean scores rather than summing all items into a single total score.

1. AI literacy score: Mean of AIL1, AIL2, AIL3, AIL4, AIL5, AIL6

2. Risk perception score: Mean of RP1, RP2, RP3, RP4

3. Academic confidence score: Mean of AC1, AC2, AC3, AC4

# Missing-data rule

If at least 80% of the items within a subscale are present, compute the subscale mean from available items.

Otherwise, set the subscale score to missing.

Applying this rule gives:

AI literacy: at least 5 of 6 items required

Risk perception: at least 3 of 4 items required

Academic confidence: at least 3 of 4 items required

# Reverse scoring

No reverse-scored items are included in the current AIRPAC-Q version.

# Variable names for scoring

AI literacy: ail1 to ail6

Risk perception: rp1 to rp4

Academic confidence: ac1 to ac4
