# Supplementary material for: Development and validation of the AI literacy, risk perception, and academic confidence questionnaire for Chinese pre-service teachers
Source: PLoS One. 2026 Jul 16;21(7):e0353837. doi: 10.1371/journal.pone.0353837 (PMC13375133; doi:10.1371/journal.pone.0353837)
Supplement: S2 Table — (DOCX) [file pone.0353837.s002.docx]

**S2 Table. Item sources, expert review summary, pilot revision decisions, and content validity indices for the AIRPAC-Q items.**

This table documents the 18-item initial pool, the basis of item wording, expert-review and pilot-test decisions, and content validity indices for retained items. Deleted candidate items are summarized by construct and deletion reason rather than included in the scoring version of the questionnaire.

| **Initial item code** | **Construct** | **Basis of item wording** | **Expert/pilot feedback and decision** | **Retained code** | **I-CVI** |
| --- | --- | --- | --- | --- | --- |
| AIL1 | AI literacy | Adapted from AI literacy literature; rewritten for education | Retained after minor wording check | AIL1 | 1.00 |
| AIL2 | AI literacy | Adapted from AI literacy/evaluation literature | Retained; emphasized reliability of AI-generated information | AIL2 | 0.83 |
| AIL3 | AI literacy | New item based on AI error/bias awareness | Retained; wording clarified to include errors or bias | AIL3 | 1.00 |
| AIL4 | AI literacy | Adapted from AI use in learning contexts | Retained; wording focused on learning support | AIL4 | 0.83 |
| AIL5 | AI literacy | New item based on limitations of AI in education | Retained after expert review | AIL5 | 0.83 |
| AIL6 | AI literacy | Adapted from critical evaluation of AI systems | Retained; wording focused on AI-generated suggestions | AIL6 | 1.00 |
| RP1 | Risk perception | New item based on perceived risk in education | Retained; broad risk statement retained as domain indicator | RP1 | 0.83 |
| RP2 | Risk perception | Adapted from misinformation/misleading-content concern | Retained; wording limited to learner impact | RP2 | 1.00 |
| RP3 | Risk perception | New item based on caution in academic reliance | Retained; central caution item | RP3 | 1.00 |
| RP4 | Risk perception | Adapted from negative consequence/risk perception literature | Retained; wording linked to learning consequences | RP4 | 0.83 |
| RP5 | Risk perception | Candidate item on privacy, bias, and integrity | Deleted after expert review because wording was double-barreled | Deleted | N/A |
| RP6 | Risk perception | Candidate item on general uncertainty about AI | Deleted after pilot testing because of redundancy with RP1 and RP3 | Deleted | N/A |
| AC1 | Academic confidence | Adapted from academic behavioural confidence; rewritten for AI-supported environments | Retained; wording specifies academic judgments | AC1 | 1.00 |
| AC2 | Academic confidence | Adapted from learning decision confidence | Retained; wording specifies using AI tools | AC2 | 0.83 |
| AC3 | Academic confidence | New item based on adapting to AI-related academic tasks | Retained after wording revision | AC3 | 0.83 |
| AC4 | Academic confidence | Adapted from self-regulated learning confidence | Retained; wording specifies managing learning when AI tools are involved | AC4 | 1.00 |
| AC5 | Academic confidence | Candidate item on general confidence in learning | Deleted after expert review because it overlapped with general self-efficacy | Deleted | N/A |
| AC6 | Academic confidence | Candidate item on feeling confident with digital tools | Deleted after pilot testing because respondents interpreted it as general technology confidence | Deleted | N/A |

Note. I-CVI values are reported for retained items after expert review. Deleted candidate items were not included in the final content-validity summary or scoring procedure. The full retained questionnaire and scoring instructions are provided in S1 Appendix. AIRPAC-Q = AI Literacy, Risk Perception, and Academic Confidence Questionnaire; I-CVI = item-level content validity index; N/A = not applicable.
